# Ultrasoft and Ultrastretchable Wearable Strain Sensors with Anisotropic Conductivity Enabled by Liquid Metal Fillers

**DOI:** 10.3390/mi14010017

**Published:** 2022-12-21

**Authors:** Minjae Choe, Dongho Sin, Priyanuj Bhuyan, Sangmin Lee, Hongchan Jeon, Sungjune Park

**Affiliations:** 1 Department of Polymer-Nano Science and Technology, Department of Nano Convergence Engineering, Jeonbuk National University, Jeonju 54896, Republic of Korea; 2 Sustainable Materials Research Team, Research & Development Division, Hyundai Motor Group, Uiwang 16082, Republic of Korea

**Keywords:** liquid metal elastomers, soft and stretchable electronics, wearable strain sensors, soft robotics

## Abstract

Herein, ultrasoft and ultrastretchable wearable strain sensors enabled by liquid metal fillers in an elastic polymer are described. The wearable strain sensors that can change the effective resistance upon strains are prepared by mixing silicone elastomer with liquid metal (EGaIn, Eutectic gallium-indium alloy) fillers. While the silicone is mixed with the liquid metal by shear mixing, the liquid metal is rendered into small droplets stabilized by an oxide, resulting in a non-conductive liquid metal elastomer. To attain electrical conductivity, localized mechanical pressure is applied using a stylus onto the thermally cured elastomer, resulting in the formation of a handwritten conductive trace by rupturing the oxide layer of the liquid metal droplets and subsequent percolation. Although this approach has been introduced previously, the liquid metal dispersed elastomers developed here are compelling because of their ultra-stretchable (elongation at break of 4000%) and ultrasoft (Young’s modulus of <0.1 MPa) mechanical properties. The handwritten conductive trace in the elastomers can maintain metallic conductivity when strained; however, remarkably, we observed that the electrical conductivity is anisotropic upon parallel and perpendicular strains to the conductive trace. This anisotropic conductivity of the liquid metal elastomer film can manipulate the locomotion of a robot by routing the power signals between the battery and the driving motor of a robot upon parallel and perpendicular strains to the hand-written circuit. In addition, the liquid metal dispersed elastomers have a high degree of deformation and adhesion; thus, they are suitable for use as a wearable sensor for monitoring various body motions.

## 1. Introduction

Soft and stretchable electronics have gained a great attention due to their potential applications in wearable sensors [1,2], soft robotics [3,4], and electronics skins [5,6]. The ability to change electric behavior of the soft and stretchable devices in response to various external stimuli can be utilized for designing the wearable sensors with targeted sensing mechanism such as piezoelectric, piezocapacitive, and piezoresistive. Among them, the piezoresistive sensors that can change the electrical resistance upon deformation of the devices have been widely utilized for measuring external strain, pressure, and forces straight forwardly [7,8].

Stretchable and soft piezoresistive sensors that can undergo a high degree of deformation can be utilized for wearable strain sensors, which are appealing for application in biomedical devices [9,10] and soft robotics [11,12] for monitoring human motion and utilizing human–machine interfaces. They can be created using elastic polymers embedded with conductive fillers and exhibit changes in electrical resistance in response to external strains [13,14]. These wearable sensors typically require mechanically competent circuits to electrically interconnect and mechanically support electronic components [15,16]. To fabricate soft and stretchable circuits, electrodes such as metal particles [17,18], metal wires [19,20], and carbonaceous fillers [21,22] have been included in elastic components; however, these conductors may compromise the mechanical softness of the elastic components owing to the rigidity of the materials.

Liquid metals (gallium and gallium alloys) are considered compelling electrodes for creating soft and stretchable circuits because they can preserve electrical conductivity while strained [23,24]. The liquid metals can form a thin (~3 nm) surface oxide layer in air [25,26]; thus, the metal can adhere to various substrates and can be patterned into desired shapes by injection [27,28], vacuum-assisted capillary filling [29,30], printing [31,32], and forced wetting [33,34]. An alternative method to form the liquid metal circuits is to utilize the liquid metal particles rendered by ultrasonication [35,36,37]. Liquid metal can break into micro- and nanosized particles in elastomers by ultrasonication and are subsequently stabilized by the oxide. Although the liquid metal particles with tunable sizes enabled by various loading volumes can benefit for use in dielectric elastomers [38,39], electronic ink [36,40,41], and catalysts [42,43], the liquid metal particle-included elastomer composites are electrically nonconductive because of the oxide layer on the particles [44,45]. Thus, various methods have been explored to restore electrical conductivity by rupturing the oxide layer of the particles and coalescing into conductive pathways. Laser- and thermal sintering of the liquid metal particles can result in conductive metallic circuits [46,47] and applying dielectrophoresis can also directly guide the liquid metal particles to align and to form the circuits [48,49]. Although these approaches can be used to pattern the metallic circuits, localizing mechanical pressure onto the liquid metal elastomer to rupture the oxide layer on the liquid metal particles is the probably most convenient method for creating circuits that can be utilized for stretchable and soft electronics because these approaches are fairly facile and can enable to create electric circuit non-lithographically, thus, it is economically favorable [50,51,52,53,54,55].

In this study, we demonstrate stretchable and soft wearable strain sensors enabled by liquid metal fillers in an elastic polymer. Once the oxide layer of the liquid metal droplets is ruptured by localizing mechanical pressure on the elastic polymer, a handwritten conductive trace is formed. Although this concept has been introduced by several groups [50,53,56], we clarify the novel aspects of the present study by demonstrating anisotropic conductivity of the liquid metal elastomer film upon parallel and perpendicular strains to the handwritten conductive trace. The handwritten circuit can typically provide an electrical pathway in the plane direction; however, we observed the varied electrical behavior of the liquid metal circuits under parallel and perpendicular strain along the direction of the conductive traces and we termed this ‘anisotropic conductivity.’ This behavior can be realized by utilizing a silicone elastomer (ExSil 100) that exhibits ultrahigh elongation at break (4000%) and ultrasoft properties (Young’s modulus of <0.1 MPa) owing to non-crosslinked and highly entangled polymeric chains. We utilized this principle to manipulate the locomotion of a robot by routing the power signals between the battery and the driving motor of a robot upon parallel and perpendicular strains to the handwritten circuit. The elastomer used in this study is ultrasoft and readily deformable in response to external strains. Thus, we utilized the liquid metal elastomer as a stretchable and soft wearable strain sensor to monitor various human motions by attaching it to curvilinear body surfaces. These ultrastretchable and soft wearable sensors with anisotropic conductivity enabled by liquid metal fillers would be useful for application in soft robotics, artificial skins, and stretchable and soft electronics.

## 2. Materials and Methods

### 2.1. Fabrication of Liquid Metal Elastomer Films

The liquid metal elastomer composites were prepared by shear mixing silicone elastomers (Sylgard 184 from Dow Corning and ExSil 100 from Gelest, Morrisville, PA, USA) and liquid metal (eutectic gallium-indium alloy, Indium Corporation, Clinton, New York, NY, USA) with various wt% using a planetary mixer at 800 rpm for 10 min (Thinky mixer ARE-310, Sotokanda, Chiyoda-ku, Tokyo, Japan). Once the silicone elastomer and liquid metal was completely mixed, the liquid metal elastomer films were prepared by thermal curing at 60 °C for 4 h, and localized mechanical pressure (normal force of 0.1 MPa, which was measured by dividing the maximum weight multiple and the gravitational acceleration by the area) was applied onto the liquid metal elastomer films using a stylus with a diameter of 8 mm and subsequently scratched along the surface of the elastomer to create conductive traces. Copper tapes were attached to both ends of the conductive traces to characterize their electrical behavior.

### 2.2. Characterization

The mechanical properties of the liquid metal elastomer films were characterized using an extensometer (Quasar 2.5 Single column, Galdabini, Cardano al Campo, Italy) with a constant load application of 1 kN at an extension rate of 10 mm/min until the samples failed. All electrical characterizations were performed using a benchtop multimeter (Keysight 34461a, Keysight Technologies, Santa Rosa, CA, USA). By connecting a benchtop multimeter and extension rate of 10 mm/min by extensometer, data was collected to evaluate how resistance changed as a function of strain. The morphology of liquid metal elastomers according to the liquid metal content was observed using an optical microscopy (Olympus CX23, Olympus Corporation, Tokyo, Japan).

## 3. Results and Discussion

**Figure 1:**
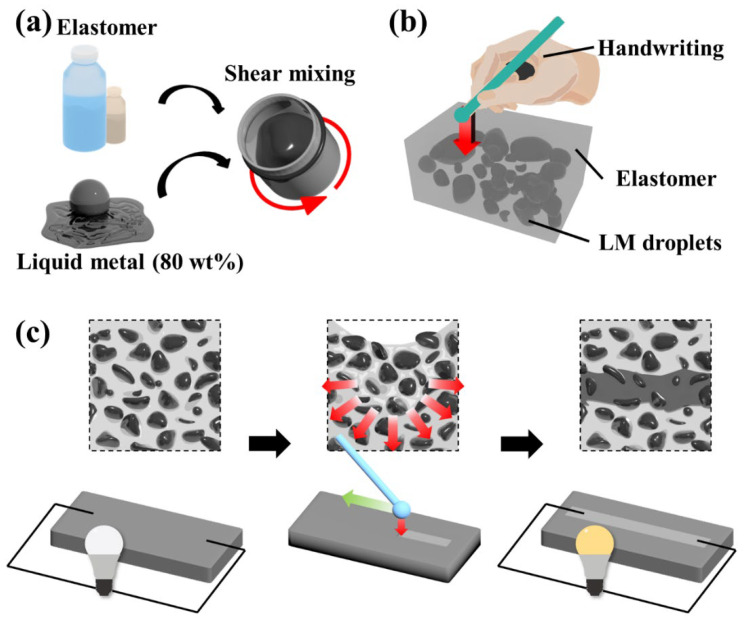
Schematics showing the fabrication process of the liquid metal elastomer film with a hand-written circuit. (**a**) Shear mixing a silicone elastomer with the liquid metal, (**b**) thermally cured silicone elastomer with the liquid metal droplets dispersed, and (**c**) conductive trace by sintering the liquid metal droplets via applying localized mechanical pressure onto the liquid metal elastomer film.

We used two silicone elastomers (Sylgard 184 and ExSil 100) to fabricate liquid metal elastomer films with conductive traces created by hand writing, as shown in Figure 1a,b. The liquid metal elastomer films were prepared by shear mixing the silicone elastomers with liquid metal (LM) fillers with different loading amounts. Once the liquid metal elastomer film was formed by shear mixing, followed by thermal curing, the LM droplets stabilized by the oxides were dispersed in the elastomeric matrix. The diameter of the LM droplets can be manipulated by varying the mixing conditions, such as the concentration of the LM and rotating speed during mixing [44,57]. The liquid metal elastomer film did not initially exhibit electrical conductivity owing to the presence of a thin oxide layer on the LM droplet surface, however, localizing mechanical pressure onto the liquid metal elastomer films resulted in rupturing of the oxide layer and percolating the liquid metal to form a conductive trace (Figure 1c). Previously, the high rigidity-induced high energy dissipation coefficient of the polymeric matrix with LM droplets can help to generate mechanically induced conductive traces by applying localized forces onto the polymer [58]. Although the elastomer used in this study is soft (Young’s modulus of 3.9 and 0.1 MPa for Sylgard 184 and ExSil 100, respectively) [59], we note that the liquid metal elastomer film can also be directly and conductively traced by multiple applications of localized mechanical pressure.

**Figure 2:**
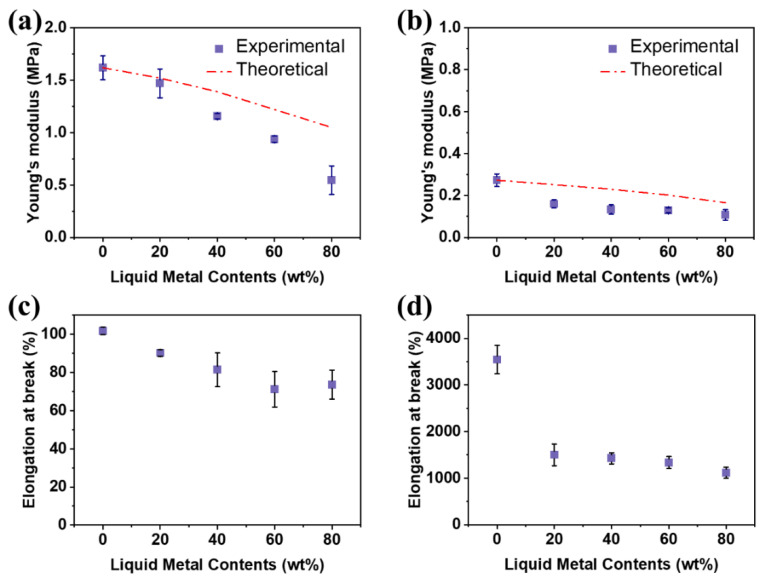
Mechanical properties of the liquid metal elastomer films made of Sylgard 184 (**a**,**c**) and ExSil 100 (**b**,**d**). (**a**,**b**) Young’s modulus of the liquid metal elastomer films as a function of the contents of liquid metal fillers. (**c**,**d**) Plot of elongation at break of the liquid metal elastomer films as a function of the contents of the liquid metal fillers.

The liquid metal elastomer films with various ratios of the LM fillers were used for characterizing the mechanical properties. Figure 2a,b shows Young’s modulus of the elastic films with the LM according to the content of the LM fillers. As the liquid metal content increases in the range of 0 to 80 wt%, the Young’s modulus of the Sylgard 184 films with the liquid metals (Sylgard 184-LM) and the ExSil 100 film with the liquid metals (ExSil 100-LM) decreases from 1.6 MPa to 0.5 MPa and from 0.3 MPa to 0.1 MPa, respectively, due to fluidic nature of the LM fillers [39,51,52,55]. The presence of fluid droplets with a high surface tension (around 360 mN m^−1^) [60,61,62,63] can affect the Young’s modulus of elastomer composites as predicted by Equation (1).

(1) $$E_{c}=E\left\{\frac{1+\frac{5\gamma }{2ER}}{\frac{5\gamma }{2ER}\left(1-\varphi \right)+\left(1+\frac{5\varphi }{3}\right)}\right\}$$

where, *E_c_*, *E*, *γ*, φ, and *R* represent the Young’s modulus of composite, Young modulus of encasing elastomer, surface tension of the LM droplet (360 mN m^−1^), volume percent (φ) of the LM droplet and the radius of the LM droplet. The experimental results are almost identical to theoretical values, i.e., difference is less than 0.6 MPa. As shown in Figure 2c,d, elongation at break of the elastomer films with the LM fillers also decrease inversely as a function of the contents of the LM fillers presumably due to large area of surface oxide of the LM droplets.

**Figure 3:**
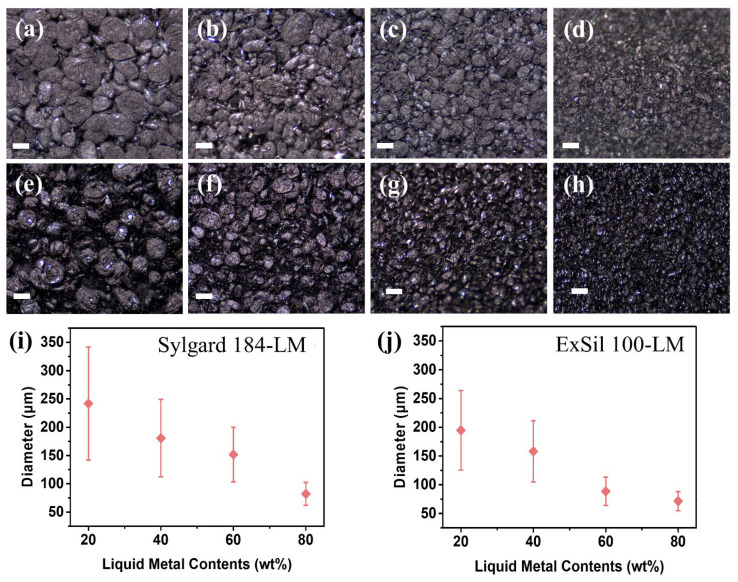
Optical microscopy images showing (**a**–**d**) the Sylgard 184 and (**e**–**h**) the ExSil 100 with the various contents of the liquid metal fillers. (**a**) 20 wt%, (**b**) 40 wt%, (**c**) 60 wt%, and (**d**) 80 wt% of the liquid metal fillers. The scale bar represents 200 μm. (**i**,**j**) The diameters of the liquid metal droplets dispersed in (**i**) Sylgard 184 and (**j**) ExSil 100 according to various contents of the liquid metal fillers.

As previously studied, Young’s modulus of the elastic composites can be changed by various diameters of the fillers [38,39,64,65]. As shown in the optical microscopy images of the elastomer composites with LM fillers (Figure 3), the diameter of the LM droplets dispersed in the elastomers decreases inversely as a function of the contents of the LM fillers due to the viscosity of the composite increased as a function of LM fillers content [12,38,44]. As the viscosity of the composite increases, more force is applied to maintain the constant rotational speed (800 rpm), resulting in the smaller radius LM droplets.

**Figure 4:**
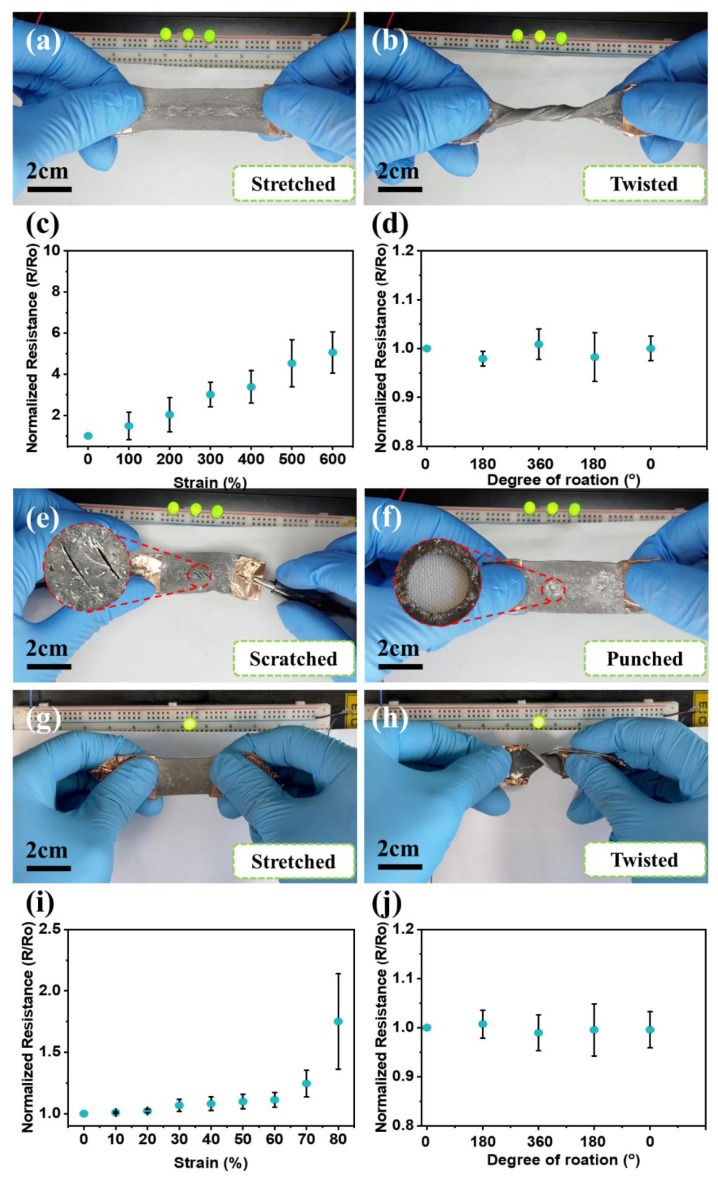
Photographs of (**a**–**f**) the ExSil 100-LM films and (**g**–**j**) the Sylgard 184-LM films showing electrical conductivity while strained. The ExSil 100-LM films were subjected to (**a**) stretching, (**b**) twisting and folding, (**e**) scratching, and (**f**) punching. (**c**,**d**) The effective normalized resistance of the ExSil 100-LM films upon (**c**) stretching and (**d**) twisting. The Sylgard 184-LM films were also subjected to (**g**) stretching and (**h**) twisting. (**i**,**j**) The effective normalized resistance of the Sylgard 184-LM films upon (**i**) stretching and (**j**) twisting.

As shown in Figure 4a–h, the liquid metal elastomer films (ExSil 100-LM and Sylgard 184-LM) with the handwritten circuits shows the electrical conductivity maintained upon various deformations, such as stretching, folding, and twisting as demonstrated by LED activation (Video S1, Supplementary Materials). The elastomer-LM films exhibits the effective resistance increased as a function of strain in parallel direction along the conductive trace (Figure 4c,i) as theoretically expected by the well-known equation R = ρ(L/A), where R denotes the effective resistance and *ρ* is the bulk resistivity of the liquid metal. As the film is stretched, the length of the trace (L) increases, whereas the cross-sectional area (A) narrows, resulting in increased resistance (R). However, it is observed that there is a little change in the effective resistance of the handwritten liquid metal circuit upon twisting because the length and area of the conductive trace is almost constant (Figure 4d,j). We also scratched the conductive trace region of the liquid metal elastomer film and punched it to make a hole with 3 mm in diameter to demonstrate the electrical conductivity of the handwritten circuit after mechanical damages without leakage owing to the liquid metal electrode stabilized by the oxides (Figure 4e,f).

**Figure 5:**
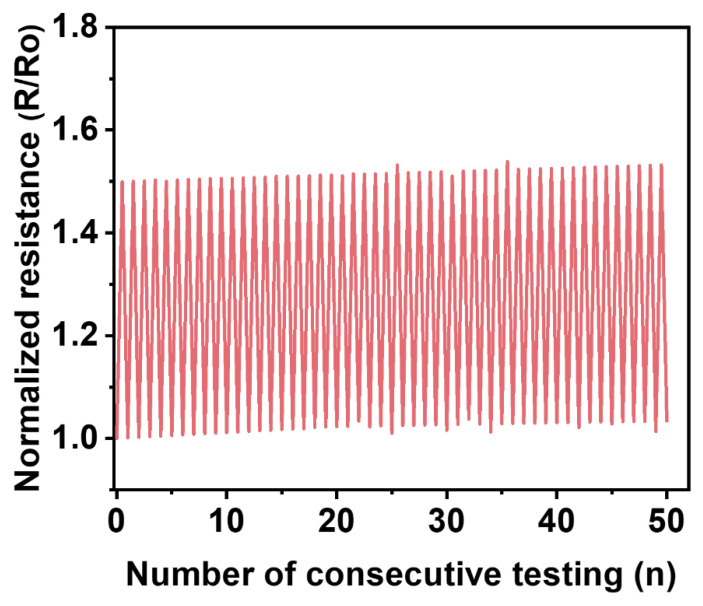
Normalized effective resistance of the liquid metal elastomer film with handwritten circuit during 50 consecutive cycles of tensile testing at strains of 100%.

We also characterized electrical stability of the liquid metal elastomer films as shown in Figure 5. The normalized resistance increased by 3% over 50 cycles of tensile testing at 100% strain. Although the fluctuation of the effective resistance increased because of the less entangled polymeric network upon consecutive tensile strains, the normalized effective resistance value increased slightly, thereby demonstrating the electrical stability of the handwritten conductive trace.

**Figure 6:**
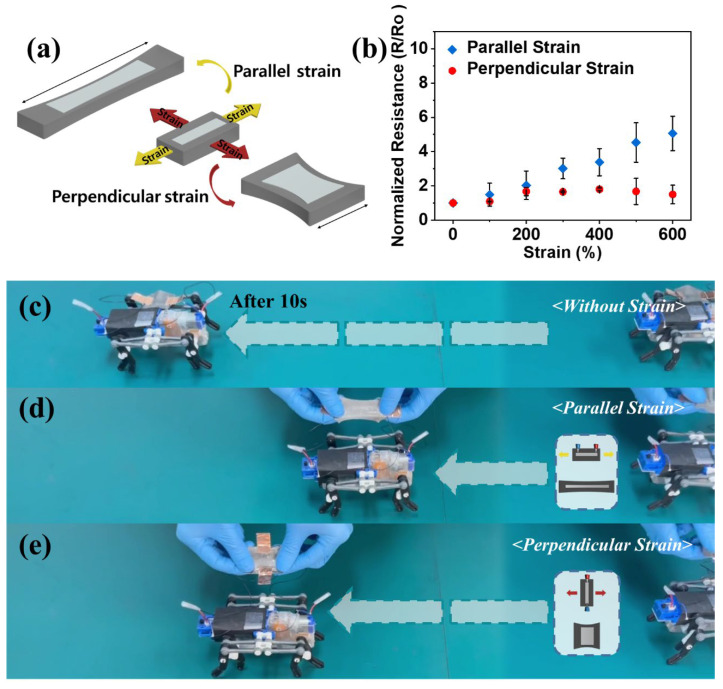
Liquid metal elastomer films exhibiting anisotropic conductivity. (**a**) Schematic showing the geometrical change of the liquid metal elastomer film with handwritten circuit upon strains parallel and perpendicular to the direction of the conductive trace. (**b**) Normalized resistance versus strain for the conductive trace upon parallel and perpendicular strains. (**c**–**e**) Photographs showing the locomotion of a robot enabled by the liquid metal elastomer film connected (**c**) without strain, (**d**) with parallel strain along the direction of the conductive trace, and (**e**) with perpendicular strain along the direction of the conductive trace.

We characterized the electrical behavior of the conductive trace when the elastic films were strained (Figure 6). We used ExSil 100-LM films due to their extremely high elongation at break induced by non-crosslinked and highly entangled polymeric network [66]. The liquid metal elastomer films can be stretched upon application of strain while maintaining its electrical conductivity (Video S1, Supplementary Materials) because of the preserved network of the percolated liquid metal electrode. As shown in Figure 6a,b, the effective electrical resistance increased as a function of strain parallel to the direction of the conductive trace because of increased length and narrowed cross-sectional area of the electrode upon strains. The effective resistance change of the conductive trace upon applying a strain of 600% parallel to the electrode was observed to be five times higher than that of the conductive trace without strain. However, the conductive trace exhibits a slightly increased electrical resistance (1.8 times) as the strain of 600% perpendicular to the electrode is applied due to the nearly constant length of the electrode. We used this anisotropic conductivity of the ExSil 100-LM upon strains to manipulate the locomotion of a robot enabled by routing the power signals between the battery and the driving motor of a walking robot as shown in Figure 6c–e. The robot rapidly moves when powered on due to the electrical conductivity of the LM circuit without strains (Figure 6c and Video S2, Supplementary Materials); however, locomotion can be manipulated by strain along the direction of the conductive trace. When the ExSil 100-LM is strained parallel to the LM electrode trace, the robot moves slowly owing to increased effective resistance upon strain (Figure 6d). However, the locomotion gets enhanced as the ExSil 100-LM is strained perpendicular to the LM electrode (Figure 6e).

**Figure 7:**
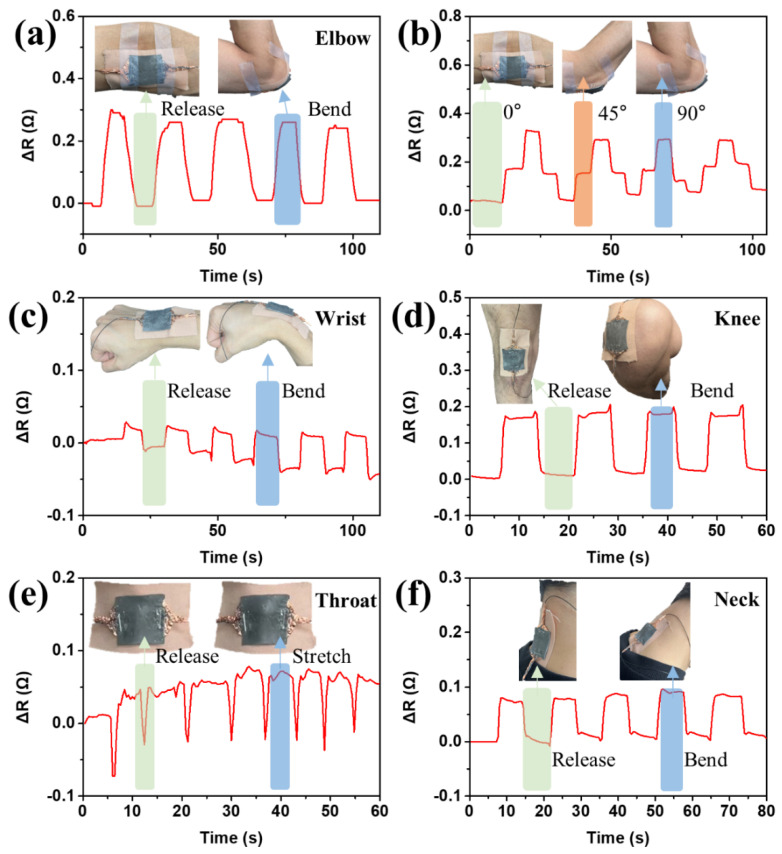
Liquid metal elastomer films utilized soft and wearable strain sensors monitoring various body motions by attaching them to the (**a**,**b**) elbow, (**c**) wrist, (**d**) knee, (**e**) throat, and (**f**) neck.

The liquid metal elastomer films can be utilized as soft and wearable strain sensors to monitor various human motions, as shown in Figure 7. The elastomer used in this study has a high degree of deformation and adhesion to the surfaces due to highly entangled polymeric network and a small amount of uncured silicone oil that prevents the polymer from adhering to the curvilinear surfaces; thus, it is suitable for use as wearable strain sensors by direct attachment to the human body. As shown in Figure 7a, the liquid metal elastomer film attached to the elbow exhibited change in the effective resistance induced by mechanical bending. A higher bending angle generated higher change in the effective resistance (Figure 7b). Similarly, liquid metal elastomer film based wearable strain sensors can change effective resistance caused by movement in joints when they are attached to the wrist, knee, throat, and neck (Figure 7c–f). The liquid metal elastomer film is strained by the movement of the uvula on the throat, thus demonstrating the sensing ability of the LM conductive trace to monitor tiny body motions (Figure 7e).

## 4. Conclusions

In summary, we demonstrated ultrastretchable and soft wearable strain sensors with anisotropic conductivity enabled by liquid metal fillers. The liquid metal elastomer film was prepared by shear mixing the elastomer with liquid metal fillers, followed by thermal curing. When the liquid metal elastomer film was mixed with liquid metal, the metal was rendered into small droplets stabilized by an oxide, resulting in a non-conductive elastomer film. However, the liquid metal elastomer film can store electrical conductivity by rupturing the oxide layer of the liquid metal droplets and sintering them by applying localized pressure using a stylus to the liquid metal elastomer film. The handwritten conductive trace in the liquid metal elastomer film can maintain the conductivity when strained; however, the conductivity is anisotropic upon strains parallel and perpendicular to the conductive trace. We utilized the anisotropic conductivity of the circuits to manipulate the locomotion of a robot by routing the power signals between the battery and the driving motor of a walking robot. The liquid metal elastomer film has a high degree of deformation and adhesion to the surface; thus, it can be utilized as a soft and wearable strain sensor to monitor various body motions. The ultrastretchable and soft liquid metal elastomer films developed in this study could be used in soft robotics, stretchable electronics, and wearable devices.

## Data Availability

The data presented in this study are available on request from the corresponding author.

## Supplementary Materials

The following supporting information can be downloaded at: https://www.mdpi.com/article/10.3390/mi14010017/s1, Video S1: Liquid metal elastomer film showing electrical conductivity while strained. Video S2: Manipulated locomotion of a robot enabled by liquid metal elastomer film with anisotropic conductivity.
